# The effects of PPARγ agonist rosiglitazone on neointimal hyperplasia in rabbit carotid anastomosis model

**DOI:** 10.1186/1749-8090-7-57

**Published:** 2012-06-20

**Authors:** Mehmet Guzeloglu, Buket Reel, Soner Atmaca, Alper Bagrıyanık, Eyup Hazan

**Affiliations:** 1 Department of Cardiovascular Surgery, Faculty of Medicine, İzmir University, Izmir, Turkey; 2 Department of Pharmacology, Faculty of Pharmacy, Ege University, Izmir, Turkey; 3 Department of Histology and Embriology, Faculty of Medicine, Dokuz Eylul University, Izmir, Turkey

**Keywords:** Neointima, Rosiglitazone, Matrix metalloproteinases (MMPs), Rabbit

## Abstract

**Background:**

Neointimal hyperplasia involving smooth muscle cell (SMC) proliferation, migration and extracellular matrix (ECM) degradation is an important component of atherosclerosis. It develops as a response to vascular injury after balloon angioplasty and vascular graft placement. Matrix metalloproteinases (MMPs) induce SMC proliferation, migration and contribute to intimal hyperplasia by degrading ECM. PPARγ agonists inhibit SMC proliferation, migration and lesion formation. In this study, we aimed to investigate the effects of PPARγ agonist rosiglitazone on neointimal hyperplasia and gelatinase (MMP-2 and MMP-9) expressions in rabbit carotid anastomosis model.

**Methods:**

New Zealand white rabbits (n = 13, 2.7–3.2 kg) were divided into placebo and treatment groups. Right carotid artery (CA) was transected and both ends were anastomosed. Treatment group (n = 6) received rosiglitazone (3 mg/kg/day/p.o.) and placebo group (n = 7) received PBS (phosphate buffered saline, 2.5 ml/kg/day/p.o.) for 4 weeks postoperatively. After the sacrification, right and left CAs were isolated. Morphometric analyses and immunohistochemical examinations for gelatinases were performed.

**Results:**

Intimal area (0.055 ± 0.005 control vs 0.291 ± 0.020 μm^2^ anastomosed, p < 0,05) and index (0.117 ± 0.002 control vs 0.574 ± 0.013 anastomosed, p < 0,01) significantly increased in anastomosed arteries compared to control arteries from placebo group. However, in rosiglitazone-treated group, intimal area (0.291 ± 0.020 PBS vs 0.143 ± 0.027 rosiglitazone, p < 0,05) and index (0.574 ± 0.013 PBS vs 0.263 ± 0.0078 rosiglitazone, p < 0,01) significantly decreased. Furthermore, gelatinase immunopositivity was found to have significantly increased in anastomosed arteries from placebo group and decreased with rosiglitazone treatment.

**Conclusions:**

These results suggest that rosiglitazone may prevent neointimal hyperplasia, which is the most important factor involved in late graft failure, by inhibiting gelatinase enzyme expression.

## Background

Neointimal hyperplasia has a major role in early restenosis after surgical interventions such as surgical revascularisation, percutan transluminal angioplasty (PTA) and stenting [1,2]. It is an early and essential step in the pathogenesis of atherosclerosis and restenosisis. This step is characterised by extracellular matrix (ECM) degradation, and medial vascular smooth muscle cell migration to intima and their proliferation.

Matrix metalloproteases (MMPs) are a family of zinc-dependent enzymes which induce smooth muscle proliferation and migration by degrading ECM and contribute to intimal hyperplasia, inflamation and plaque rupture [3]. Therefore, inhibition of MMPs may be a crucial strategy to reduce the development of intimal hyperplasia.

Peroxisome proliferator-activated receptors (PPARs) are ligand-activated nuclear receptor family. The three PPAR isotypes, PPAR-α, PPAR-δ and PPAR-γ modulate the function of many target genes and participate in the regulation of vital processes such as inflammation, cell growth, proliferation, migration and differentiation [4-6].

PPARγ is expressed predominantly in adipose tissue and it is also present in endothelial cells, smooth muscle cells and monocytes/macrophages [7]. Recent studies demonstrated that the activation of PPARγ inhibited MMP expression in cultured macrophages and hypercholesterolemic mice [8,9]. This effect contribute to their antiproliferative effect on SMCs. Indeed, PPARγ agonists are shown to decrease migration and proliferation of human and rat vascular SMCs [5]. Similarly, it was reported that dominant-negative loss of PPARγ function enhances SMC proliferation, migration, and vascular remodeling in isolated transgenic mice SMCs [10].

Thiazolidinediones (TZDs), which are widely used in the treatment of type II diabetics as insulin sensitizers, are selective activators of PPARγ [11]. Rosiglitazone, a synthetic PPARγ agonist, was reported to inhibit neointimal hyperplasia in rats after balloon injury, and to reduce SMC proliferation in rat SMC culture [5,12,13]. Moreover, clinical studies revealed that PPARγ agonists, rosiglitazone and pioglitazone inhibit development of neointimal hyperplasia and restenosis after percutaneus coronary intervention in diabetic coronary artery patients [14,15].

Although vascular protective effects of rosiglitazone in some atherosclerosis models and cell culture are known, its effects on proinflammatory gelatinase A and B enzymes (MMP-2 and MMP-9) related to atherosclerotic process were not thoroughly understood.

In the light of the collected data, the purpose of the present study was to investigate the effects of PPARγ agonist rosiglitazone on neointimal hyperplasia process and gelatinase expressions in rabbit carotid anastomosis model.

## Methods

### Animals

This study was approved by the Local Ethics Committee of Dokuz Eylul University, School of Medicine. All animals received care in compliance with the principles of laboratory animal care formulated by the National Society for Medical Research and the Guide for the Care and Use of Laboratory Animals. In this study, New Zealand white rabbits of either sex (n = 13; 2,7 – 3,2 kg) were used. Rabbits were randomly divided into two groups as placebo and treatment groups. Throughout the 4-week treatment period, rabbits from treatment group (n = 6) received rosiglitazone (3 mg/kg/day, p.o.) postoperatively [16]. Rabbits from placebo group (n = 7) received only the vehicle (PBS; phosphate buffered saline) (2.5 ml/kg/day, p.o.) for the same period. Throughout the 4-week treatment period each rabbit was kept in a separate cage and allowed to access to regular diet (standard rabbit chow and tap water ad libitum). All animals tolerated drug treatment well. The treatment protocol did not affect survival rate and body weight of animals from both groups (data not shown).

### Surgical procedures

Rabbits were anesthetized with intramuscular xylazine (3 mg/kg) and ketamine (50 mg/kg). All procedures were performed by the same surgeon using a 3.5X surgical telescope (US PAT NO: 3273456, Designs for Vision, Ronkonkoma, NY, USA). An oblique cervical incision was made and the right carotid artery was surgically accessed. Rabbits were heparinised with 100 IU/kg heparin. Three minutes after heparisination, distal and proximal parts were clamped and the right carotid artery was transected. Subsequently, both ends of right carotid artery were anastomosed in an end-to-end form using an 8/0 polypropylene suture [17]. Then, a similar incision was made on the left contralateral carotid artery and a segment from the left carotid artery which constituted control groups (Rosiglitazone control and PBS control) was resected. Afterwards, the carotid arteries were returned to their original positions and the anatomic layers were closed properly. Following the operation xylazine was given intramusculary to the rabbits for analgesia.

On the 28^th^ postoperative day, the rabbits were sacrificed using an over dose of sodium pentobarbital (150 mg/kg). Subsequently, the anastomosed right carotid artery segment and then a segment from the contralateral intact carotid artery as control artery were isolated and removed. Each segment was put into a tube containing 10% neutral buffered formalin immediately for histological examination.

### Histological evaluations

#### Morphometry

After sacrification of the rabbits, tissue samples were prepared for analysis by light microscopy and immunohistochemistry. Tissues were fixed in 10% neutral formalin solution for 24–48 hrs. Then the samples were embedded in paraffin and serial sections were taken onto glass slides as in 5 μm thickness. After staining by Orcein Light Green, they were mounted by using Entellan mounting medium. Samples were examined under a light microscope (Olympus BH2, Tokyo, Japan) and images were transferred to the computer using a digital video camera (Olympus DP71, Tokyo, Japan). The light microscope was connected to an image capture system in all groups and measurements. Multiple fields (up to 5) were selected from each section of each sample in a systematic random pattern and images were taken and analyzed by using software (Image Tool, Uthscsa-version 3.00 for Windows, analysis system). The images were processed and measurements were taken in cross-sections. Briefly, intima and media areas (μm^2^) were meausured using computer-assisted image analyzer system. Additionally, ratios of intima/media (index values) were calculated.

#### Immunohistochemistry

Five micrometer sample sections were incubated at 60°C overnight then deparaffinised in xylene for 30 min. After rehydrating by using a decreasing series of ethanols, sections were washed in distilled water for 10 min. Then they were heated with 10 mM citrate buffer (Cat # AP-9003-125 Labvision) for five minutes, to unmask antigens. After washing in deionized water for three times with two minutes period, sections were delineated using a Dako pen (Dako, Glostrup, Denmark), In order to inhibit endogenous peroxidase activity samples were incubated in 3% H2O2 for 5 min. After blocking samples with serum solution for 30 minutes, sections were incubated with primary antibody in a humid chamber for 18 h at +4°C. Anti-MMP-2 (cat. # MAB 3308, Milipore, MA, ABD), anti-MMP-9 (Cat. # MAB 3309, Milipore, MA, ABD) primary antibodies were used. Samples were then incubated with anti-mouse secondary antibodies conjugated with biotin and with streptavidin conjugated to horseradish peroxidase for 30 min each prepared according to kit instructions (Cat # 85–9043, Histostain-Plus Bulk Kit Broad Spectrum, Invitrogen, Camarillo, California, USA). They were finally incubated with 3,30 diaminobenzidine hydrochloride (DAB) (Cat # 1718096, Roche, Oenzberg, Germany), and nuclei were counterstained with Mayer’s hematoxylin. All dilutions and washing steps were performed by using phosphate buffered saline (PBS), pH 7.4. Sections were dehydrated through a graded ethanol series, cleared in xylene, mounted in Entellan (Cat # 107961, Merck, Darmstadt, Germany) and analyzed using a light microscope. Appropriate positive controls were also stained.

### Statistical analysis

Statistical analysis of morphometric data were performed for drug treatments (two levels, rosiglitazone or placebo) as between rabbit factor; and anastomosis (two levels, present or not) as within rabbit factor with factorial analysis of variance (ANOVA). If there were interactions between the factors in ANOVA, Wilcoxon signed ranks test and Mann Whitney U test were used for paired and unpaired comparisons respectively.

Chi-square test was used to evaluate the statistical difference of immunohistochemical data between rosiglitazone and placebo group. Statistical analyses of paired data (anastomosed vs control) from immunoscoring were performed with Wilcoxon signed ranks test. Shown are means ± SEM. n indicates the number of animals. p < 0.05 was considered statistically significant.

## Results

### Morphometry

Intense intimal hyperplasia was observed in anastomosed right arteries as compared to those in control left arteries in placebo group as a result of morphometric examinations.

**Figure 1:**
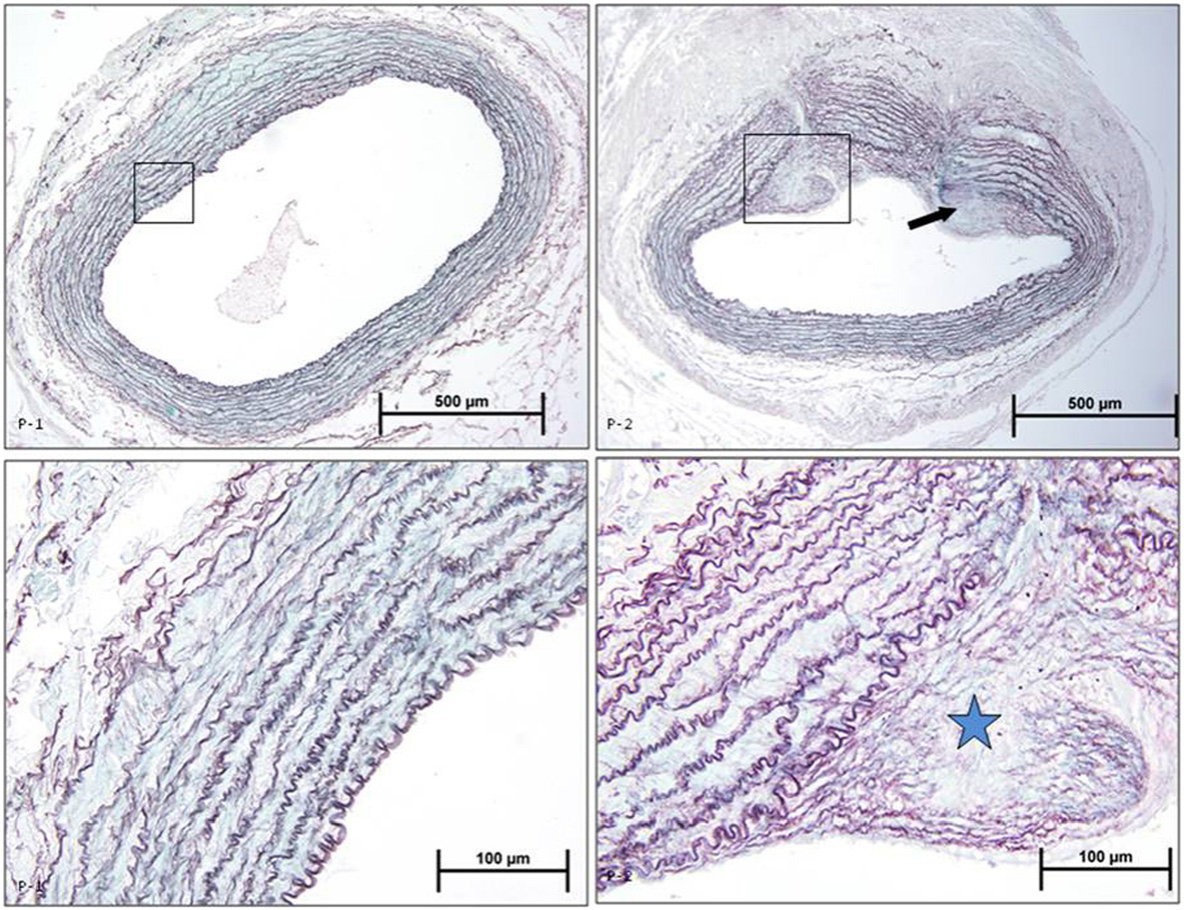
**Photomicrographs of orcein light green stained rabbit carotid arteries.** P1: Control left artery from PBS group, P2: Anastomosed right artery from PBS group. Star and arrow indicate intimal hyperplasia in the artery.

**Figure 2:**
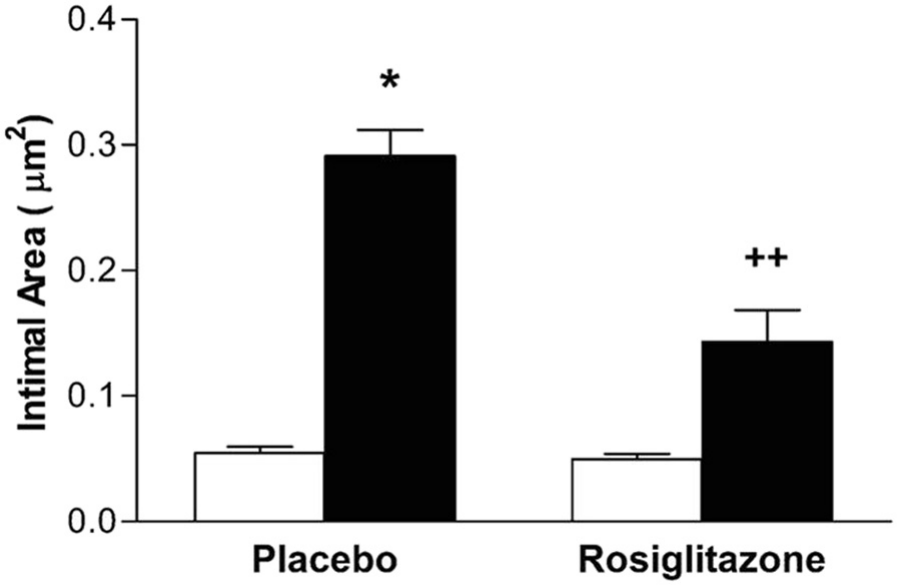
**Effects of anastomosis and rosiglitazone on intimal hyperplasia in rabbit carotid arteries.** White columns represent control left, black columns represent anastomosed right carotid arteries. n represents number of the rabbits. Placebo group (n = 7), rosiglitazone group (n = 6). *p < 0.05 Wilcoxon signed ranks test (control left vs anastomosed right arteries) and ++p < 0.01 Mann Whitney U test (placebo vs rosiglitazone groups).

**Figure 3:**
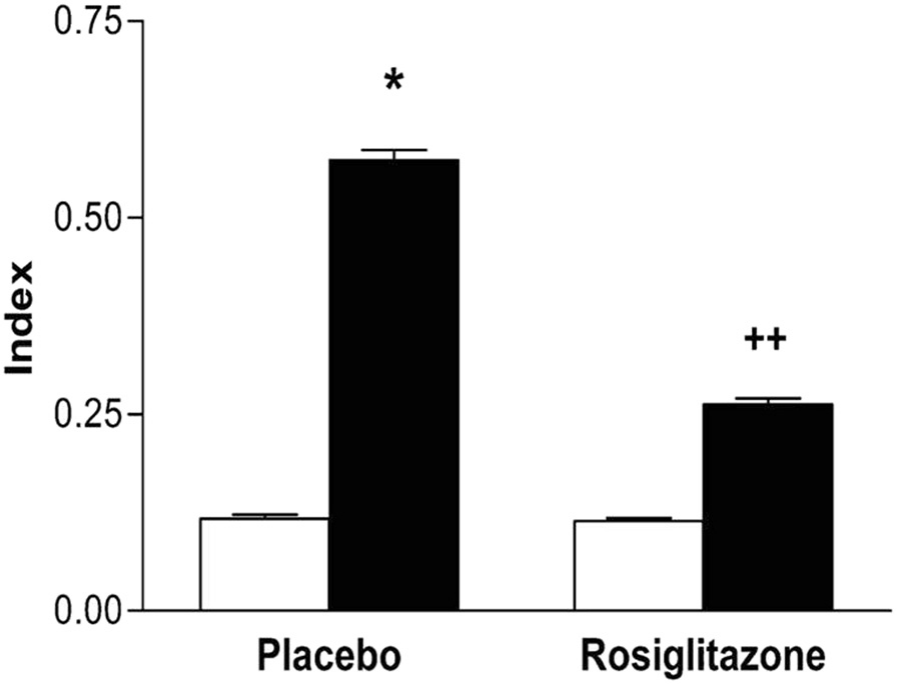
**Effects of anastomosis and rosiglitazone on index in rabbit carotid arteries.** White columns represent control left, black columns represent anastomosed right carotid arteries. n represents number of the rabbits. Placebo group (n = 7), rosiglitazone group (n = 6). *p < 0.05 Wilcoxon signed ranks test (control left vs anastomosed right arteries) and ++p < 0.01 Mann Whitney U test (placebo vs rosiglitazone groups).

The intimal cross-sectional area (0.055 ± 0.005 μm^2^ control left vs 0.291 ± 0.020 μm^2^ anastomosed right arteries) (p < 0.05) and the ratio of intimal area to medial area (index) (0.117 ± 0.002 control vs 0.574 ± 0.013 anastomosed right arteries) (p < 0.05) significantly increased in anastomosed right arteries as compared to those in control left arteries in placebo group (Figure 1; Figure 2; Figure 3).

**Figure 4:**
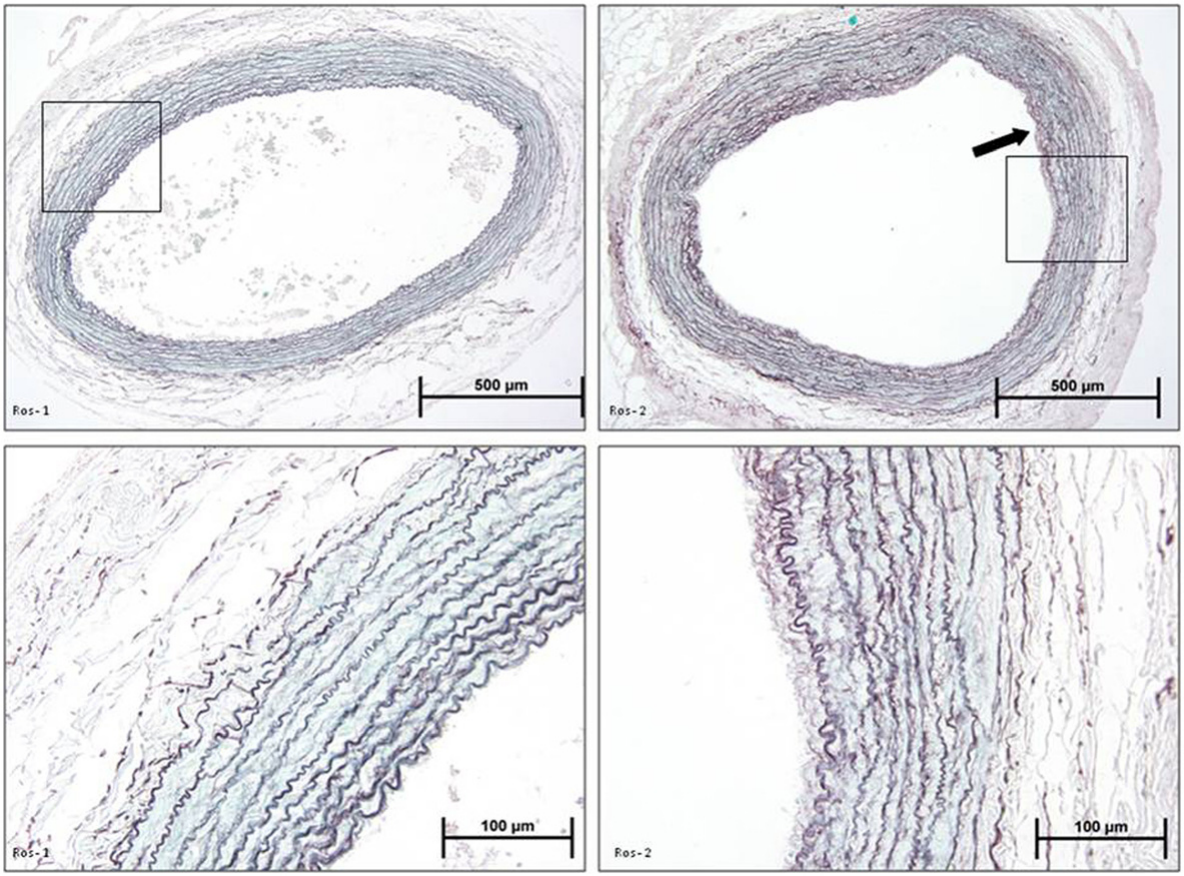
**Photomicrographs of orcein light green stained rabbit carotid arteries.** ROS1: Control left artery from rosiglitazone group, ROS2: Anastomosed right artery from rosiglitazone group. Arrow indicates intimal hyperplasia in the artery.

However, rosiglitazone treatment significantly decreased the neointimal hyperplasia (0.291 ± 0.020 μm^2^ PBS group vs 0.143 ± 0.027 μm^2^ rosiglitazone group) (p < 0.01) and carotid artery intima/media index (0.574 ± 0.013 PBS group vs 0.263 ± 0.0078 rosiglitazone group) (p < 0.01) in anastomosed right arteries (Figure 2, Figure 4, Figure 3).

Medial area (0.508 ± 0.032 μm^2^ control left vs 0.468 ± 0.043 μm^2^ anastomosed right arteries) (p = 0.075) and luminal area (0.444 ± 0.042 μm^2^ control left vs 0.579 ± 0.072 μm^2^ anastomosed right arteries) (p = 0.137) did not change in anastomosed right arteries in the placebo group respectively. Rosiglitazone treatment did not affect either the medial (0.540 ± 0.094 μm^2^ PBS group vs 0.435 ± 0.038 μm^2^ rosiglitazone group) (p = 0.999) or luminal cross-sectional areas (0.487 ± 0.085 μm^2^ PBS group vs 0.545 ± 0.074 μm^2^ rosiglitazone group) (p = 0.953) respectively in anastomosed right arteries.

### Immunohistochemistry

**Figure 5:**
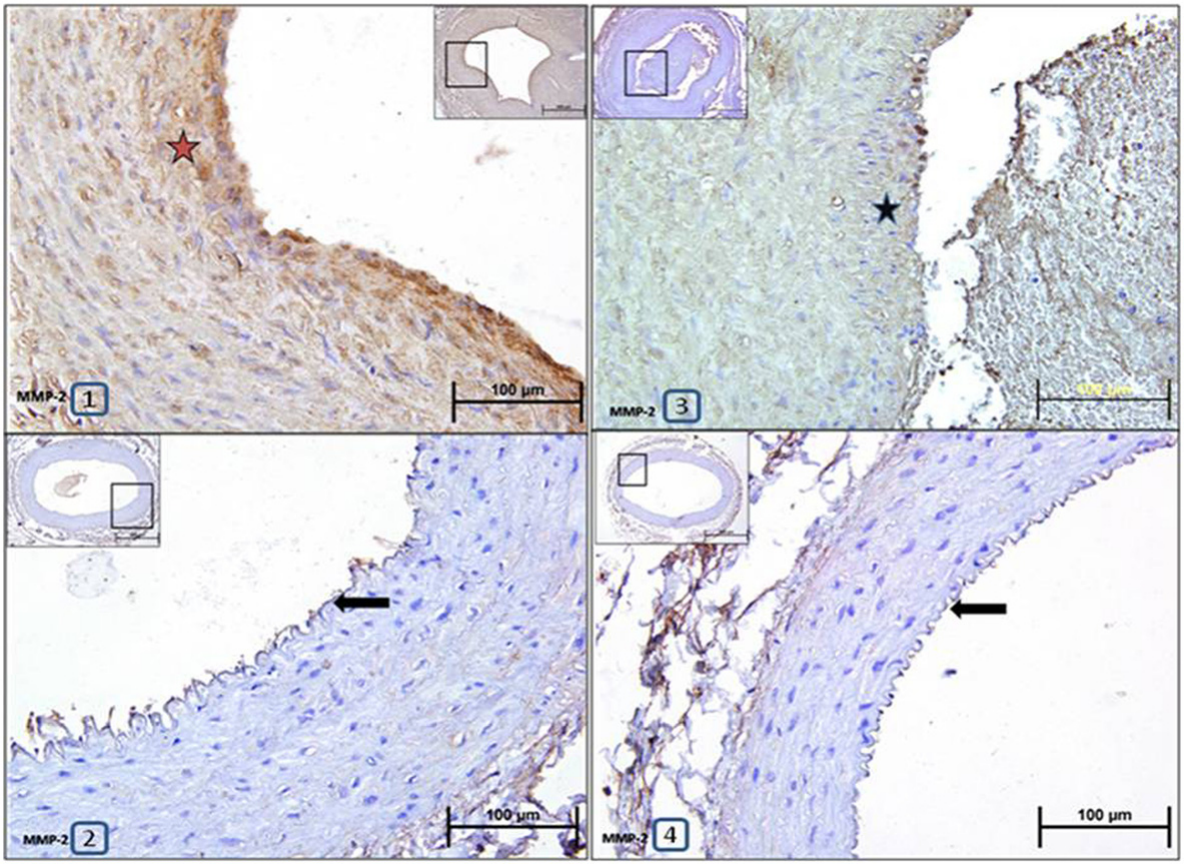
**Representative photomicrographs of paraffin transverse sections of carotid arteries stained with MMP-2 antibody immunohistochemically.** 1:Anastomosed right carotid artery from placebo group, 2:Control left carotid artery from placebo group, 3:Anastomosed right carotid artery from rosiglitazone-treated group, 4:Control left carotid artery from rosiglitazone-treated group. Stars show brown stained MMP-2 immunopositive areas in the arteries. Arrows point out intima layers of the arteries.

**Figure 6:**
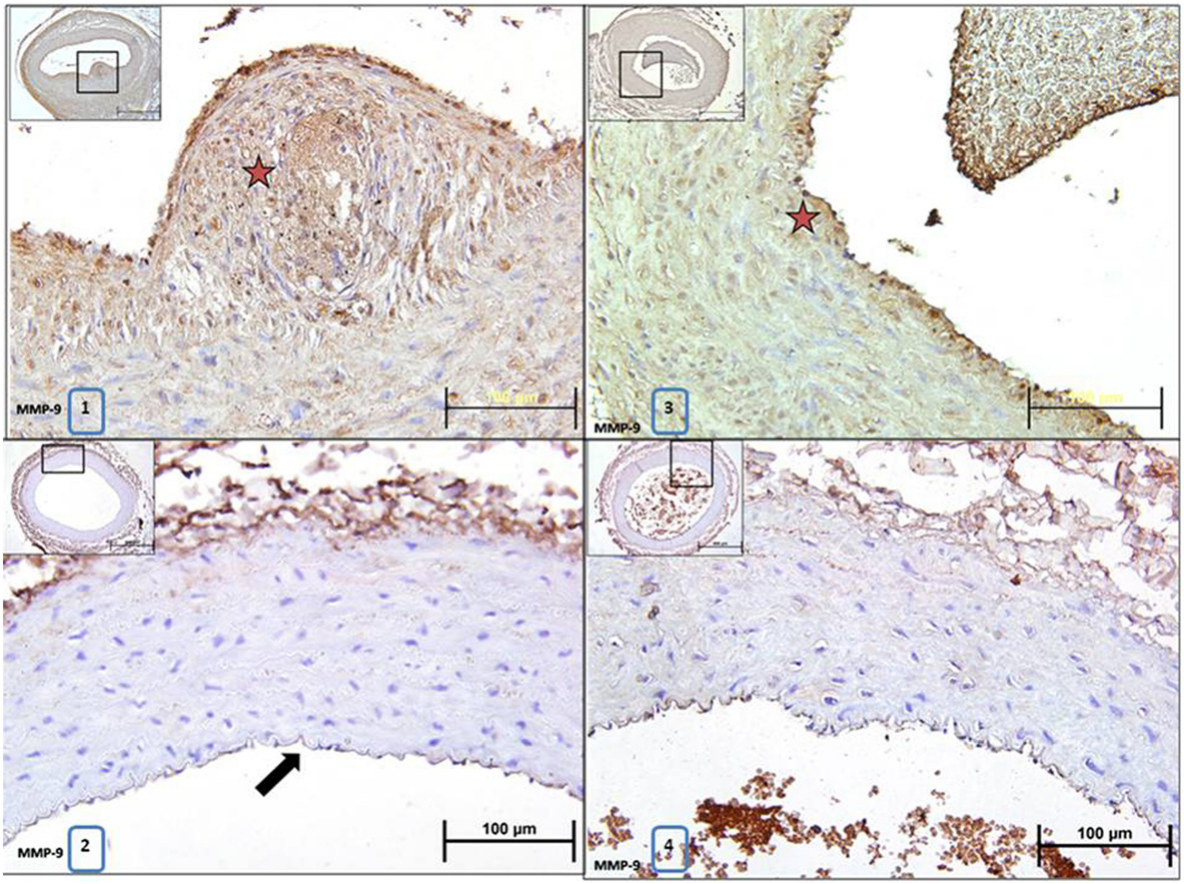
**Representative photomicrographs of paraffin transverse sections of carotid arteries stained with MMP-9 antibody immunohistochemically.** 1:Anastomosed right carotid artery from placebo group, 2:Control left carotid artery from placebo group, 3:Anastomosed right carotid artery from rosiglitazone-treated group, 4:Control left carotid artery from rosiglitazone-treated group. Stars show brown stained MMP-9 immunopositive areas in the arteries. Arrow points out intima layer of the artery.

Immunoscoring with MMP-2 and MMP-9 antibodies revealed that immunopositivity for MMP-2 and MMP-9 significantly increased in anastomosed right arteries compared to control left arteries from placebo group (Figure 5, Table 1). However, immunoscoring for MMP-2 and MMP-9 showed that rosiglitazone treatment significantly decreased immunopositivity for these two proteins in anastomosed right carotid arteries compared to placebo group (Figure 6, Table 1).

**Table 1:** İ**mmuno scores for MMP-2 and MMP-9 in both anastomosed right and control left carotid arteries from placebo (n = 7) and rosiglitazone-treated (n = 6) groups**

Immunoscores	0	1	2	3
MMP-2				
Placebo Left	7	0	0	0
PlaceboRight*	0	0	1	6
Rosiglitazon Left	6	0	0	0
Rosiglitazon Right ++	0	4	2	0
MMP-9				
Placebo Left	7	0	0	0
Placebo Right*	0	0	2	5
Rosiglitazon Left	6	0	0	0
Rosiglitazon Right ++	0	5	1	0

## Discussion

The primary reason for late bypass graft failure is neointimal hyperplasia which occurs in response to vascular injury [18,19]. In the present study we showed that carotid artery anastomosis model leads to intensive neointimal hyperplasia and increased intima/media index in rabbits from placebo group consistent with our previous findings [20]. However, luminal and medial areas remained unchanged in anastomosed arteries compared to control arteries from placebo group as we have observed previously in our study [20]. This latter finding may suggest that there is a expansive vascular remodeling to compensate narrowed luminal area and to maintain luminal patency in anastomosed arteries. Vascular remodeling is an adaptive changing and healing process in arterial size and composition and also determines the degree of luminal narrowing in vascular occlusive diseases, restenosis and atherosclerosis [21,22]. Expansive vascular remodeling notably occurs during the early period of graft adaptation and protects luminal area [23]. However, we have not aimed to examine remodeling process in the arteries in this study. This point needs further investigation.

In the present study, we also demonstrated that rosiglitazone inhibited neointimal hyperplasia induced by carotid artery anastomosis model in rabbits without affecting luminal or medial area. Previous reports have pointed out that PPAR-γ receptor agonists could prevent restenosis and atherosclerosis [14,24]. Similarly, in a recent study, PPAR-γ receptor ligand rosiglitazone was shown to decrease neointima and increase luminal area of the vessels after balloon catheter injury in collesterol-fed rabbits [25]. Besides, it was reported that rosiglitazone reduced inflammatory response and retarded neointima progression after vascular injury in experimental atherosclerosis models in mouse and rats [26]. Furthermore, rosiglitazone was demonstrated to protect from restenosis and atherosclerosis after vascular injury in diabetic animals, and after coronary stent location in diabetic patients [14,24,27]. In all these models, anti-atherosclerotic effects of rosiglitazone seem to be pleiotropic effects independent of their effects on glucose and lipid metabolism [28]. However, the underlying mechanisms of these pleiotropic effects are not clearly known. Therefore, we investigated the possible role of gelatinases and effects of rosiglitazone in our experimental atherosclerosis model in the current study. As parallel to our previous findings, we demonstrated semiquantitatively that carotid artery anastomosis model in rabbits induces gelatinase upregulation associated with neointimal hyperplasia formation [20]. We have observed increasing trend of gelatinase expression in this model previously. Unlikely, in the present study, we determined the increased expressions of two gelatinase enzymes semiquantitatively by scoring in rabbits [20].

Previous studies showed that unbalanced increase in MMP activity after vascular injury may cause various vascular pathologies including atherosclerosis, plaque rupture, restenosis and vein graft failure [3,29,30]. In particular, gelatinases (MMP-2 and MMP-9) were shown to be implicated in early atherosclerotic process [29,31]. It was reported that SMC migration from intima to media and induction of intimal hyperplasia substantially decreased in deficiency of gelatinases [32,33]. Consistent with our findings, recent studies demonstrated that gelatinases increased after vascular injury in femoral artery wire injury model in murins, and vascular graft or collar induced atherosclerosis models in rabbits [23,34,35]. Furthermore, increased gelatinase expression was shown in balloon-injured Sprague–Dawley rats and aortic smooth muscle cell culture, and also human saphenous vein organ culture from patients undergoing coronary bypass surgery [36,37]. Additionally, it was reported that administration of an MMP inhibitor after ballon injury or overexpression of TIMP-1 and TIMP-2 or inhibitors of gelatinases, respectively, inhibited neointima formation in vivo [38-40]. Our results support all these previous evidences concerning gelatinases and further underline the major role of gelatinases, and significance of ECM breakdown, promoting proliferation and migration of vascular SMCs in atherosclerotic process.

Additionally, the increase in the levels of pro-inflammatory gelatinases in the anastomosed arteries points out the presence of inflammation after vascular injury in our model. It was known that inflammatory cytokines such as interleukin-1 and −4 (IL-1 and IL-4) and tumour necrosis factor-α (TNF-α) may coordinately induce gelatinase enzymes during progression of atherosclerosis [30]. Moreover, growth factors such as platellet derived growth factor (PDGF) and fibroblast growth factor-2 (FGF-2) could interact with these cytokines [3]. Furthermore, chemokines may direct transendotelial migration of monocytes and differentiation to macrophages. In the next step, monocytes and macrophages could cause increased MMP expression [41]. All these possible effects were shown to contribute to SMC proliferation and migration from intima to media layer, and finally intimal hyperplasia [3,30]. However, further studies are necessary to elucidate the underlying molecular signaling mechanisms of increased MMP expression in this model.

As another key finding, in the current study we have demonstrated for the first time that rosiglitazone significantly inhibited gelatinase expression in carotid artery anastomosis model in rabbits. Indeed, it was known that PPAR-γ receptor agonists have important role in the modulation of vital processes such as inflammation, proliferation, differentiation and apoptosis independent of their actions in metabolic control [6,28]. In addition, Wu et al. demonstrated that LPS-induced gelatinase A (MMP-2) activity was inhibited by rosiglitazone in the rat aortic endothelial cells [42]. In agreement with this study, Lee et al. showed that rosiglitazone inhibited the expression and activity of gelatinase B (MMP-9), and vascular SMC proliferation and migration through GSK-3β activation and inhibition of NF-kB and ERK pathways in rat aortic vascular SMC culture [13]. Furthermore, Rinaldi et al. showed that rosiglitazone diminished neointimal hyperplasia and inflammatory events by reducing numbers and interactions of inflammatory cells and also negatively regulating the activations of inflammatory markers such as p38 MAPK, NF-B, COX-2, and HSP 47 [26]. Besides, Ling et al. demonstrated that rosiglitazone significantly decreased MMP-2 mRNA expression and activity, and inhibited VSMC proliferation in cultured VSMCs which were isolated from rat thoracic aortas [43]. Evidence from clinical studies are similar to the data from numerous animal studies. Clinical studies showed that patients with a history of coronary artery disease (CAD) and type 2 diabetes had significantly elevated serum MMP-9 levels [44,45]. High levels of MMP-9 were reported to trigger plaque instability and rupture [46]. However, rosiglitazone treatment decreased MMP-9 plasma levels in patients with CAD and diabetes [45]. Overexpression of MMP-9 in the human vulnerable plaques indicates the crucial role of MMP-9 in patients with acute coronary syndrome [46]. Therefore, the inhibitory effect of rosiglitazone on MMP-9 found in the present study may contribute to beneficial effects of rosiglitazone on cardiovascular risk of the patients with previous acute myocardial infarction.

## Conclusion

The present study demonstrated that PPAR-γ agonist rosiglitazone inhibited neointimal hyperplasia and expression of proinflammatory MMP enzymes, which have a crucial role in initiation and progression of atherosclerosis or restenosis in carotid artery anastomosis model in rabbits.

Our results suggest that rosiglitazone may play an atheroprotective role via its beneficial effects on gelatinases (MMP-2 and −9) throughout inflammatory process particularly in the high cardiovascular risk population of patients with diabetes mellitus. Even though some adverse effects of rosiglitazone treatment have been reported recently, there is insufficient evidence of any adverse effect on developing cardiovascular disease. Therefore, it is still used in USA with approval of FDA. However, further studies are necessary to elucidate the molecular mechanism of PPARγ agonists in vascular protection. Collected data will be useful to prove that future PPAR agonists not only prevent atherosclerotic events but also result in a net reduction on total cardiovascular incidents without any significant noncardiovascular adverse effects with long-term use.

## Competing interests

The authors declare that they have no competing interests.

## Authors’ contribution

MG, carried out the molecular genetic studies. BR, carried out the molecular genetic studies. SA, participated in the sequence alignment and drafted the manuscript. AB, participated in the sequence alignment and drafted the manuscript. EH participated in the sequence alignment and drafted the manuscript. All authors read and approved the final manuscript.
